# Supplementary material for: Cellular pharmacokinetic of methotrexate and its modulation by folylpolyglutamate synthetase and γ-glutamyl hydrolase in tumor cells
Source: PLoS One. 2024 Jun 4;19(6):e0302663. doi: 10.1371/journal.pone.0302663 (PMC11149982; doi:10.1371/journal.pone.0302663)
Supplement: Attachment — Submitted filename: Response to Reviewers.docx [file pone.0302663.s001.docx]

Dear the editor and reviewers:

Thank you very much for giving us an opportunity to revise our manuscript **PONE-D-24-04745)** titled **Cellular pharmacokinetic mechanisms of methotrexate accumulation and its modulation by folylpolyglutamate synthetase and γ-glutamyl hydrolase in tumor cells**. We appreciate the positive and constructive comments and suggestions from the editor and reviewers, which significantly improved the quality of our manuscript. We have revised the manuscript accordingly. Both the comments and the responses are listed below point by point.

Editorial office comments:

1. Please ensure that your manuscript meets PLOS ONE's style requirements, including those for file naming. The PLOS ONE style templates can be found at

https://journals.plos.org/plosone/s/file?id=wjVg/PLOSOne_formatting_sample_main_body.pdf and

https://journals.plos.org/plosone/s/file?id=ba62/PLOSOne_formatting_sample_title_authors_affiliations.pdf

Response: Thank you very much for the good advice, We have checked throughout the manuscript and revised.

2. Please note that PLOS ONE has specific guidelines on code sharing for submissions in which author-generated code underpins the findings in the manuscript. In these cases, all author-generated code must be made available without restrictions upon publication of the work. Please review our guidelines at https://journals.plos.org/plosone/s/materials-and-software-sharing#loc-sharing-code and ensure that your code is shared in a way that follows best practice and facilitates reproducibility and reuse.

Response: Thank you very much for your reminding. We have ensured the code is shared in a way that follows best practice and facilitates.

3. Thank you for stating the following in the Acknowledgments Section of your manuscript:

"This work was supported by Hunan Provincial Natural Science Foundation of China (S2017JJMSXM1571), Hunan Key Laboratory for Bioanalysis of Complex Matrix Samples (2017TP1037) and Fundamental Research Funds for the Central Universities of the Central South University (2019zzts759)"

We note that you have provided additional information within the Acknowledgements Section that is not currently declared in your Funding Statement. Please note that funding information should not appear in the Acknowledgments section or other areas of your manuscript. We will only publish funding information present in the Funding Statement section of the online submission form.

Please remove any funding-related text from the manuscript and let us know how you would like to update your Funding Statement. Currently, your Funding Statement reads as follows:

"The author(s) received no specific funding for this work."

Please include your amended statements within your cover letter; we will change the online submission form on your behalf.

Response: Sorry for this error. We have removed the fundings-related text from the manuscript. And we ensure that the authors received no specific fundings for this word.

4. We note that your Data Availability Statement is currently as follows: ""All relevant data are within the manuscript and its Supporting Information files.""

Please confirm at this time whether or not your submission contains all raw data required to replicate the results of your study. Authors must share the “minimal data set” for their submission. PLOS defines the minimal data set to consist of the data required to replicate all study findings reported in the article, as well as related metadata and methods (https://journals.plos.org/plosone/s/data-availability#loc-minimal-data-set-definition).

For example, authors should submit the following data:

- The values behind the means, standard deviations and other measures reported;

- The values used to build graphs;

- The points extracted from images for analysis.

Authors do not need to submit their entire data set if only a portion of the data was used in the reported study.

If your submission does not contain these data, please either upload them as Supporting Information files or deposit them to a stable, public repository and provide us with the relevant URLs, DOIs, or accession numbers. For a list of recommended repositories, please see https://journals.plos.org/plosone/s/recommended-repositories.

If there are ethical or legal restrictions on sharing a de-identified data set, please explain them in detail (e.g., data contain potentially sensitive information, data are owned by a third-party organization, etc.) and who has imposed them (e.g., an ethics committee). Please also provide contact information for a data access committee, ethics committee, or other institutional body to which data requests may be sent. If data are owned by a third party, please indicate how others may request data access.

Response: Thank you very much for your reminding. We have ensured the all relevant date are within the manuscript and its supporting information files.

5. PLOS requires an ORCID iD for the corresponding author in Editorial Manager on papers submitted after December 6th, 2016. Please ensure that you have an ORCID iD and that it is validated in Editorial Manager. To do this, go to ‘Update my Information’ (in the upper left-hand corner of the main menu), and click on the Fetch/Validate link next to the ORCID field. This will take you to the ORCID site and allow you to create a new iD or authenticate a pre-existing iD in Editorial Manager. Please see the following video for instructions on linking an ORCID iD to your Editorial Manager account: <https://www.youtube.com/watch?v=_xcclfuvtxQ>.

Response: Thank you very much for your reminding. We have ensured that we have an ORCID in Editorial Manager.

Reviewer #1: In this manuscript, the authors studied the metabolic process of methotrexate in cells based on the newly established UPLC -MS/MS absolute quantification method of FPGS. They established a cellular pharmacokinetic model of methotrexate and investigated the correlation between the abundance of key enzymes, FPGS and GGH, and corresponding functional parameters. Moreover, the authors elucidated the intrinsic mechanism of the phenomenon that slower infusion with more side effects. Overall, this article is informative while there still remains some problems.

1. Please note the consistency of the manuscript, there are only six curves in Figure 2 without MTX-PG6, while Table 1 has MTX-PG6, and the same problem appears in Figure 4.

Response: Thank you very much for your constructive suggestions. In our experiment.	The concentration of MTX-PG_6_ is much smaller than other metabolites, which failed to map effectively with the concentration of other metabolites, and this date has been used in other articles.

2. In section 3.2, “However, it was found in this study that most of the accumulation concentrations of MTXPG2-6 after 24 hours of gradient methotrexate treatment had no correlation with the treatment methotrexate concentration”, is there any data to support this conclusion?

Response: Thank you very much for the good advice. We added the relevant reference to support this conclusion. And the details have been provided in section 3.2.

3. In section 3.3, “There are differences in the accumulation process of MTXPG between different cell lines, and related studies have shown that this difference is related to the differential expression of methotrexate-related metabolic enzymes”, please cite relevant references.

Response: Thank you very much for the good advice. We have cited the relevant reference to support “There are differences in the accumulation process of MTX-PG between different cell lines, and related studies have shown that this difference is related to the differential expression of MTX-related metabolic enzymes”, and

details have been provided in section 3.3.

4. Please mark all abbreviations when they first appear in the text, such as “UPLC-MS”.

Response: Sorry for these errors. We have checked throughout the manuscript and revised.

5. The quality of the figures in the manuscript requires improvement (such as Figure 2). Please provide clearer, high-resolution images and ensure that all texts are legible.

Response: Sorry for these errors. We have checked and revised figures.

6. The authors may consider revising the language in the manuscript to improve the clarity and readability of the text.

Response: Thank you very much for the good advice. Our manuscript has been edited by a native English-speaking expert to ensure its English is good enough for publication. And the details have been provided in manuscript.

Reviewer #2: The manuscript“Cellular pharmacokinetic mechanisms of methotrexate accumulation and its modulation by folylpolyglutamate synthetase and γ-glutamyl hydrolase in tumor cells” established UPLC-MS/MS absolute quantification method of FPGS and GGH. However, the manuscript is lack of novelty. The whole experimental design only stays at the cellular level. I recommend to accept this manuscript after miajor revision.

(1)The English writing should be carefully edited by a native speaker of a proofread professional.

Response: Thank you very much for the good advice. Buy your suggestion, our manuscript has been edited by a native English-speaking expert to ensure its English is good enough for publication. And the details have been provided in manuscript.

(2)Fig 1 should be redrawn to make this picture more artistic.

Response: Thank you very much for the good advice. We used the colored picture to revise our manuscript, and have specific response to the dear reviewers.

Fig 1 Cellular and external MTX transport process and intracellular methotrexate metabolism process (Vmax is the saturation rate when intracellular MTX-PG_1_ is converted to PG_2-7_; k_GGH_ is the kinetic constant)

(3)Some language and grammar errors still should be carefully checked through the manuscript. In abstract, the format and language need to be modified.

Response: Thank you very much for the good advice. Buy your suggestion, our manuscript has been edited by a native English-speaking expert to ensure its English is good enough for publication. And the details have been provided in manuscript, and have specific response to the dear reviewers.

(4)The A,B,C font in fig2 and fig3 is inconsistent.

Response: Thank you very much for the good advice. We have checked and revised the figures.

(5)Some in vivo experiments need to be supplemented.

Response: Thank you very much for the nice suggestion. We deeply agree that results from in vivo experiments would enhance the depth of our research，thereby elevating the quality of our paper. However, in the present study, we mainly focus on firmed that the abundance of FPGS is the decisive factor limiting the synthesis rate of MTX-PG, and we think the present experiments may not be optimal, but should be sufficient to draw a conclusion that “ the abundance of FPGS is the decisive factor limiting the synthesis rate of MTX-PG”. We will conduct in vivo experiments as you suggested in the future work.
